# Sequestration and Transfer of Cry Entomotoxin to the Eggs of a Predaceous Ladybird Beetle

**DOI:** 10.1371/journal.pone.0144895

**Published:** 2015-12-14

**Authors:** Débora P. Paula, Lucas M. Souza, David A. Andow

**Affiliations:** 1 Embrapa Genetic Resources and Biotechnology, Parque Estação Biológica, W5 Norte, P.O. Box 02372, Brasília, DF, 70770–917, Brazil; 2 Department of Entomology, University of Minnesota, 219 Hodson Hall, 1980 Folwell Ave., St. Paul, Minnesota 55108, United States of America; University of Natural Resources and Life Sciences, Vienna, AUSTRIA

## Abstract

In the past 10 years, sequestration of Cry toxins and transfer to offspring has been indicated in three insect species in laboratory studies. This work directly demonstrates the sequestration and intergenerational transfer of Cry1F by the parents of the aphidophagous coccinellid predator, *Harmonia axyridis*, to its offspring. Recently emerged adults (10 individual couples/cage/treatment) were exposed during 20 days to aphids (100 *Myzus persicae* each day) that fed on a holidic diet containing 20 μg/mL Cry1F (and a control-group). Egg batches and neonate larvae were monitored daily, and counted and weighed for immunodetection of Cry1F by ELISA. At the end of the bioassay, the parents were weighed and analyzed by ELISA. Cry1F was detected in the offspring, both eggs and neonate larvae, of exposed *H*. *axyridis* adults. On average the neonate larvae had 60% of the Cry1F concentration of the eggs from the same egg batch. The Cry1F concentration in the adults was positively correlated with the concentration in their eggs. These three results provided independent evidence of transfer to offspring. No detrimental effects of Cry1F were observed on the age of first reproduction, total number of eggs laid per female, age-specific fecundity, egg development time, hatching rate, or fertility rate. The occurrence and generality of intergenerational transfer of Cry toxins should be investigated in the field to determine its potential ecological implications.

## Introduction

Cry toxins have been broadly used for agricultural insect pest control in genetically modified (GM) plants [1]. Presently, they are one of the most common biopesticides studied for potential environmental effects, particularly the potential effects of GM plants on beneficial insects such as biological control agents [2,3]. Nearly all of these works concentrated on the transfer of Cry toxin from the prey to the predator and did not investigate what happened to the Cry toxins in the predator themselves. For example, are Cry toxins uptaken into the predator? Does the predator sequester Cry toxins in its body? Does the predator transfer the Cry toxins to its offspring? Through these means, the predator can generate new routes of exposure and intergenerational effects that are being overlooked, even though there are some reports of uptake, sequestration and intergenerational transfer of Cry toxins in non-target insect species [4–6].

Many field studies with GM plants have detected Cry toxins in natural enemies [7–14]. However, field studies cannot distinguish transitory detection from toxin uptake. Uptake is the absorption and incorporation of a chemical into a living organism [15], and has been demonstrated in six non-target species by the continued presence of a Cry toxin after exposure has stopped and gut contents eliminated [4–6,16].

Sequestration is the deposition or storage of a chemical into specialized tissues or glands of an organism after uptake [17]. Insect natural enemies are known to sequester secondary plant metabolites [18–21], but sequestration of proteins by insects, more specifically of Cry toxins by non-target natural enemies, has just started to be observed [4,5].

Intergenerational transfer of Cry toxins involves the transfer of Cry toxin from parents to the eggs and must be preceded by uptake and sequestration. Intergenerational transfer of a Cry toxin was most clearly demonstrated in the lepidopteran *Chlosyne lacinia* [6]. Non-lethal concentrations of Cry1Ac were fed to the parental generation and transfer was confirmed by detection of Cry1Ac in eggs by western blot and higher mortality of neonates. It was also found for two more species, the coccinellid *Propylaea japonica* [4] by detection in eggs and the planthopper *Nilaparvata lugens* [5] by detection in an egg parasitoid. However, in both of these cases, the significance of the transfer was not acknowledged.

In the context of risk assessment of potential ecological effects of Cry toxins on natural enemies, it is relevant to investigate the generality of sequestration and intergenerational transfer of Cry toxins to offspring in species with a key ecological function, such as arthropod predators. Such investigations will determine the generality of extended persistence of Cry toxins in the food web, exposing other natural enemies to Cry toxins with a higher likelihood than previously considered and enabling multigenerational effects [6]. Based on its occurrence in three species in different insect orders, we hypothesized that intergenerational transfer of Cry toxins may occur generally, and we tested this in a predaceous natural enemy.

The uptake of Cry1F by larvae of the coccinellid, *Harmonia axyridis*, has been demonstrated by Paula and Andow [16] by detection of the toxin in pupae and adults after larval exposure. Larvae preyed on *Myzus persicae* aphids exposed to the toxin through an artificial diet. This voracious aphid predator is abundant in many agroecosystems with Cry1F GM crops [22], which is one of the most common GM crops worldwide. In this work we investigated if *H*. *axyridis* adults could also uptake Cry1F and, more importantly, sequester and transfer it to their offspring. We also checked for potential effects of Cry1F on the reproduction of *H*. *axyridis*. Specifically, we examined the possibility of increased Cry1F transfer to offspring and greater effects on reproduction with increased time of exposure, and we looked for parent-offspring and egg-neonate correlations in Cry1F concentrations.

## Materials and Methods

### Insects

Aphids (Hemiptera: Aphididae) were collected from various plants (such as *Bidens pilosa*, *Sonchus oleraceus*, *Tithonia diversifolia*, and *Brassica oleracea*) from private organic farms (geographical locations: -15.565148° -48.031061° and -15.611568° -48.077887°) in the Distrito Federal (Brazil) with the owner’s permission, and used to rear *H*. *axyridis*. In these farms, no GM crops or insecticides based on *Bacillus thuringiensis* were used. Adult *H*. *axyridis* (Coleoptera: Coccinellidae) were collected in the same area as the aphids and reared in plastic cages (20x15 cm) in a growth chamber at 25±2°C, 60±10% R.H. and 16L:8D. Water (offered with a wet cotton wick) and aphids were supplied daily until pupae formation. Fresh coccinellid egg batches were transferred to separate cages and checked daily to collect neonates to rear individually in plastic cups in the same growth chamber. Newly emerged adults (< 24 h old) were used in the bioassays. The collections were authorized by SISBIO (authorization number 36950), and access to the genetic heritage and transportation of biological material was authorized by IBAMA (authorization number 02001.008598/2012-42).

### Preparation of the cages

Trypsinized and purified Cry1F toxins (ca 65 kDa) was purchased from Dr. M. Pusztai-Carey (Department of Biochemistry, Case Western Reserve University, Cleveland, Ohio) and its biological activity was confirmed as described in Nakasu *et al*. [23], and summarized as follows. The biological activity of the Cry1F was verified by comparing the survival (Student’s *t*-test) after seven days of second instars of susceptible *Anticarsia gemmatalis* (Lepidoptera: Noctuidae) feeding on a solid diet with: 1) 150 μl of water containing 50 μg of Cry1F (*n* = 10); or 2) 150 μl of water (control-group, *n* = 10). The tritrophic artificial system for the predator bioassays was prepared as in Paula and Andow [24], based on Douglas & van Emden [25], and summarized as follows. The artificial system consisted of a sachet made with two layers of Parafilm M, inside of which was 300 μl of liquid holidic diet for rearing aphids [26], and attached to one end of a transparent acrylic tube (2.5 cm diameter x 2.5 cm height, wall thickness 0.35 cm). Before assembly of the system, the tube and parafilm pieces were sterilized by UV radiation for 30 min in a laminar flow hood, and the artificial liquid diet was filtered using a sterilization filter (pore size of 0.22 μm). Aphids were carefully transferred into the tubes using a paint-brush (#2).

### Cry toxin exposure bioassay

Unfed recently emerged (<24 h) female and male (*n* = 10 couples per treatment) were transferred to cages containing at least 100 *M*. *persicae*, which had fed for 24 h on a diet with and without Cry1F at 20 μg/mL. This Cry1F concentration is similar to that found in leaves of WideStrike^®^ cotton [27]. Variation in the size of the females and males was stratified across the treatments. Each cage was supplied with a wet filter paper (1 cm^2^), and cages were replaced daily with water and 100 *M*. *persicae* and inspected daily for oviposition. Eggs were washed three times with distilled water and counted under a stereomicroscope (10-20x magnification). Half of the eggs laid by a couple on a day were transferred to microtubes, weighed and stored at -20°C for ELISA analysis. The other half were kept to estimate fertility, hatching, and egg development time. Unfed neonates hatching from the egg batch laid by one couple on one day were counted, weighed and stored for ELISA analysis. Unhatched eggs were examined for embryogenesis, and eggs without an embryo were considered infertile. Fifteen egg batches were obtained from each couple in each treatment, after which adults were held for 24 h without feeding and individually weighed and stored at -20°C for ELISA analysis.

### Cry1F quantification

The Cry toxins were detected and quantified using double sandwich enzyme-linked immunosorbent assay (ELISA PathoScreen plate, Agdia, USA) according to manufacturer’s instructions. To increase the number of eggs and larvae for ELISA, the 1^st^ and 2^nd^, 3^rd^ and 4^th^, 9^th^ and 10^th^, 12^th^ and 13^th^, and 14^th^ and 15^th^ egg batches were combined, and also the neonates from the corresponding egg batches, so that there were 10 egg samples and 10 neonate samples for each control and Cry1F couple. The samples (eggs, neonates, and adults) were macerated using a glass pestle and homogenized in phosphate-buffered saline with Tween 20 (PBST) with 210 μL for the egg and unfed neonate samples, and 250 μL for each of the adults. The samples were centrifuged at 15,500x*g* for 15 min at 4°C and the supernatant used for the analysis. Each sample was applied (100 μl/well) in duplicate technical replicates. Cry1F standards at 0, 0.0625, 0.125, 0.25, 0.5, and 1.0 ng/well were applied in duplicate on each plate to estimate a linear calibration curve by regression. The absorbance was measured at 630 nm by a microtiter plate reader (TP Reader NM Thermo Plate^®^, USA).

The LODs (Limit of Detection) for Cry1F detection in the predator samples were calculated using the standard deviation and slope method and the average number of eggs, neonates or mg adult per well. The LODs were 1.51 ng/egg, 1.67 ng/neonate and 0.013 ng/mg FW of adults.

### Statistical analysis

Each ELISA plate was set up to contain multiple blanks, standards and controls that matched the egg, neonate and/or adult samples from the Cry1F treatment. The amount of Cry1F in each sample was estimated with the average slope of the calibration curves, the normalized absorbance for each sample, and the number of eggs or neonates or the weight of adults. Absorbances were normalized for each plate by subtracting the average of the blanks from the corresponding plate and then normalized for the relevant control by subtracting the control mean from the corresponding plate.

The parameters compared between the control and Cry1F treatment were male and female weights, duration of the preoviposition period (age of first reproduction), coccinellid fecundity (total number of eggs laid per female and age-specific fecundity (*m*_*x*_), see [28]), and age-specific hatching rate, fertility rate, and egg development time. In addition the concentration of Cry1F in eggs and neonates was compared to investigate the potential accumulation of Cry1F with increased exposure time. An ANCOVA was conducted to determine if Cry1F concentration in an egg batch predicted the Cry1F concentration in neonate larvae [29].

Age-specific fecundity and egg development time were analyzed by maximum likelihood repeated measures analysis using a mixed model [29] with couples as subjects, age as the repeated measure and treatment as a fixed effect. AIC_c_ was used for model selection. For age-specific fecundity, the model with first order autoregressive (AR-1) correlated errors was the best, and for development time, the model with uncorrelated errors was the best. Male and female weights, age of first reproduction, and the total number of eggs were analyzed with ANOVA [29]. Hatching and fertility were analyzed with repeated measures logistic regression (logit link, binomial error) using generalized estimating equations with QIC for model selection [29]. The residual deviance was overdispersed for both, so the scale parameter was estimated from the Pearson residual deviance. The models with uncorrelated errors were the best for both regressions. Correlations among Cry1F concentrations and reproductive parameters were calculated using Pearson’s correlation coefficient and the Fisher approximation. All input datasets are provided in S1 File. The sufficient statistics are provided in S2, S3 and S4 Files.

## Results

### Detection of Cry toxin in the offspring

**Fig 1:**
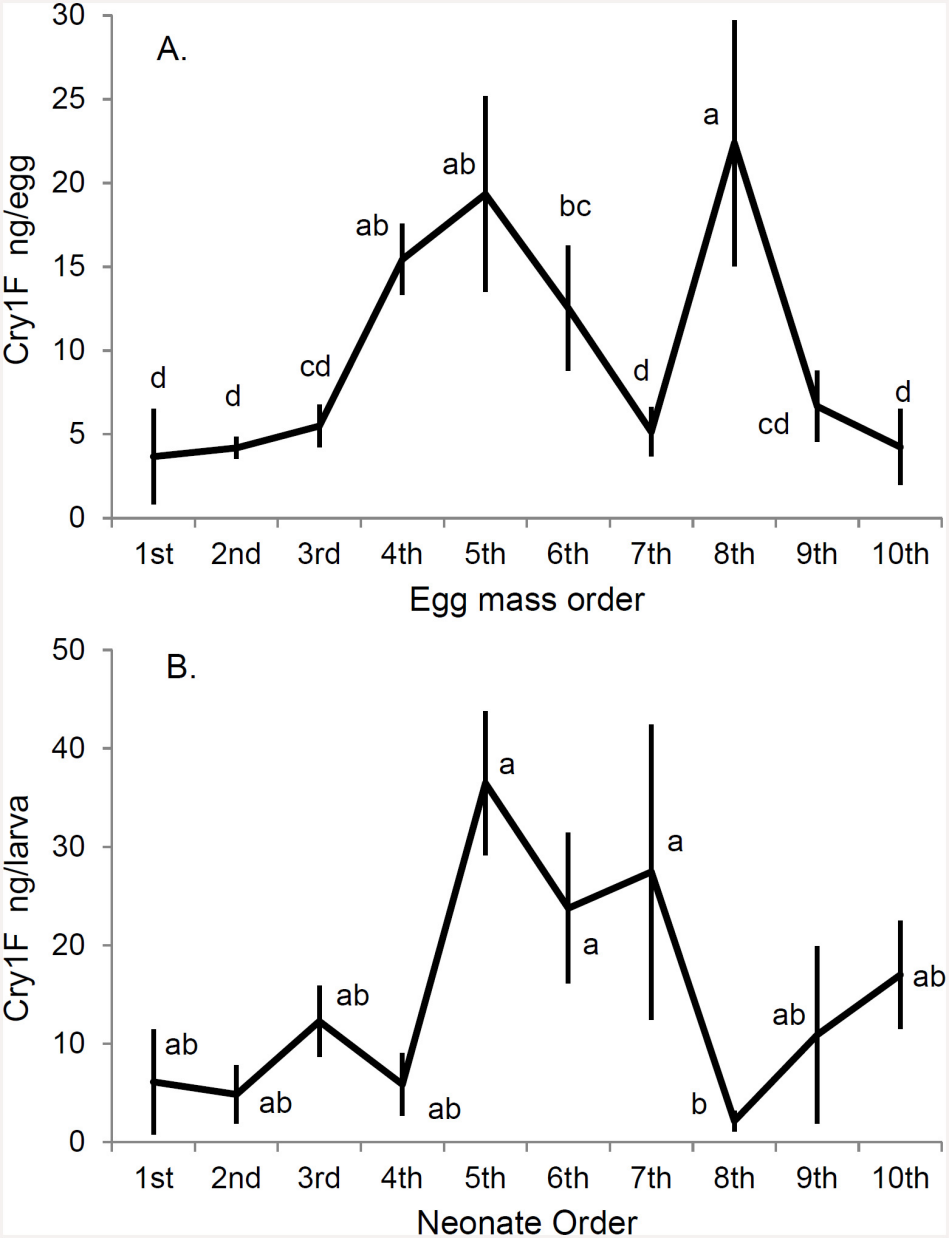
Average (±SE) Cry1F concentration (ng per offspring) in egg and neonate samples: A) First egg sample to the tenth; B) First neonate sample to the tenth. Averages are for 10 couples and averages followed by the same letter are not significantly different (Tukey’s HSD).

Cry1F was detected in eggs and larvae (Fig 1, and S2 File). In eggs, the amount of Cry1F detected varied with the order of oviposition from 3 to 22 ng/egg (*P* = 5.350x10^-6^). Egg concentrations increased during the first five samples, then leveled off, and declined in the final two samples. The first five samples occurred during the first 12 days of adult exposure, so during this early period, Cry1F concentrations increased with longer adult exposure. Neonate samples varied from 5 to 33 ng/neonate larva (*P* = 0.0297) and had higher variation than the egg samples (Fig 1B). The ANCOVA showed that Cry1F neonate concentrations were linearly related to the concentrations in the eggs from the same egg batch (*P* = 0.0300), and that neonates contained on average about 60% of the toxin concentration in the respective eggs.

**Fig 2:**
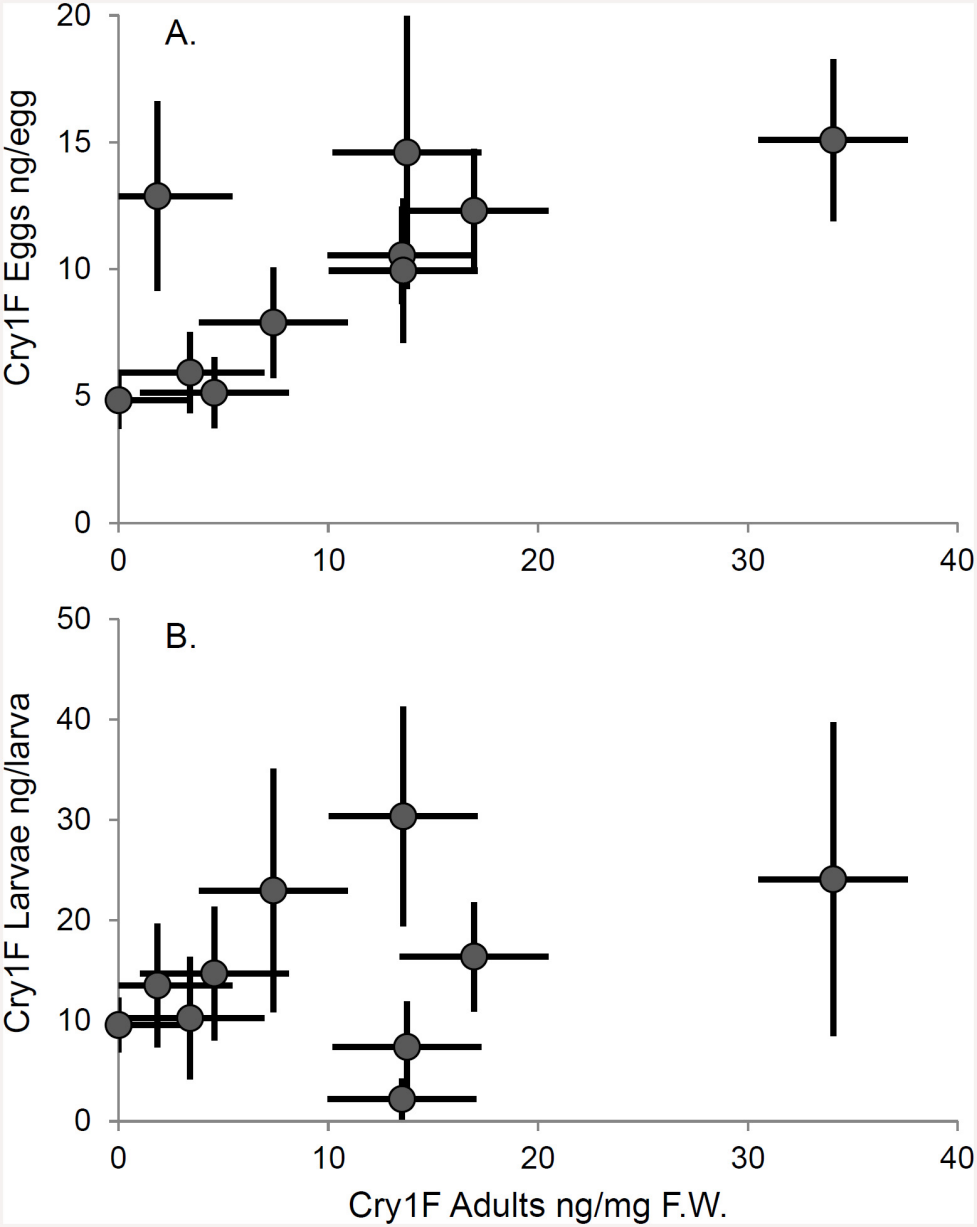
Relation between Cry1F concentration in parents (ng/mg F.W.) and the average Cry1F concentration in A) Eggs (ng/egg, *r* = 0.77; *n* = 10; *P* = 0.02) or B) Neonates (ng/neonate, *r* = 0.59; *n* = 10; *P* = 0.15). Each point is a different couple (±SE). Adults are the average of both parents. Eggs and neonates are the averages for all eggs and neonates measured for each couple.

At the end of the experiment (around 20 days), after 24 h starvation, the concentration (±SE) of Cry1F was 11.46±2.01 ng/mg F.W. in the mothers and 10.35±0.85 ng/mg F.W. in the fathers, with no significant difference between them (*P* = 0.8736; S2 File). The concentration of Cry1F in the parents was positively correlated with the concentration of Cry1F detected in their egg batches (Fig 2, *r* = 0.77; *n* = 10, *P* = 0.021), but not significantly correlated with the concentration in their neonate larvae (Fig 2, *r* = 0.59; *n* = 10; *P* = 0.155).

### Effect of Cry1F toxin on reproductive parameters

**Fig 3:**
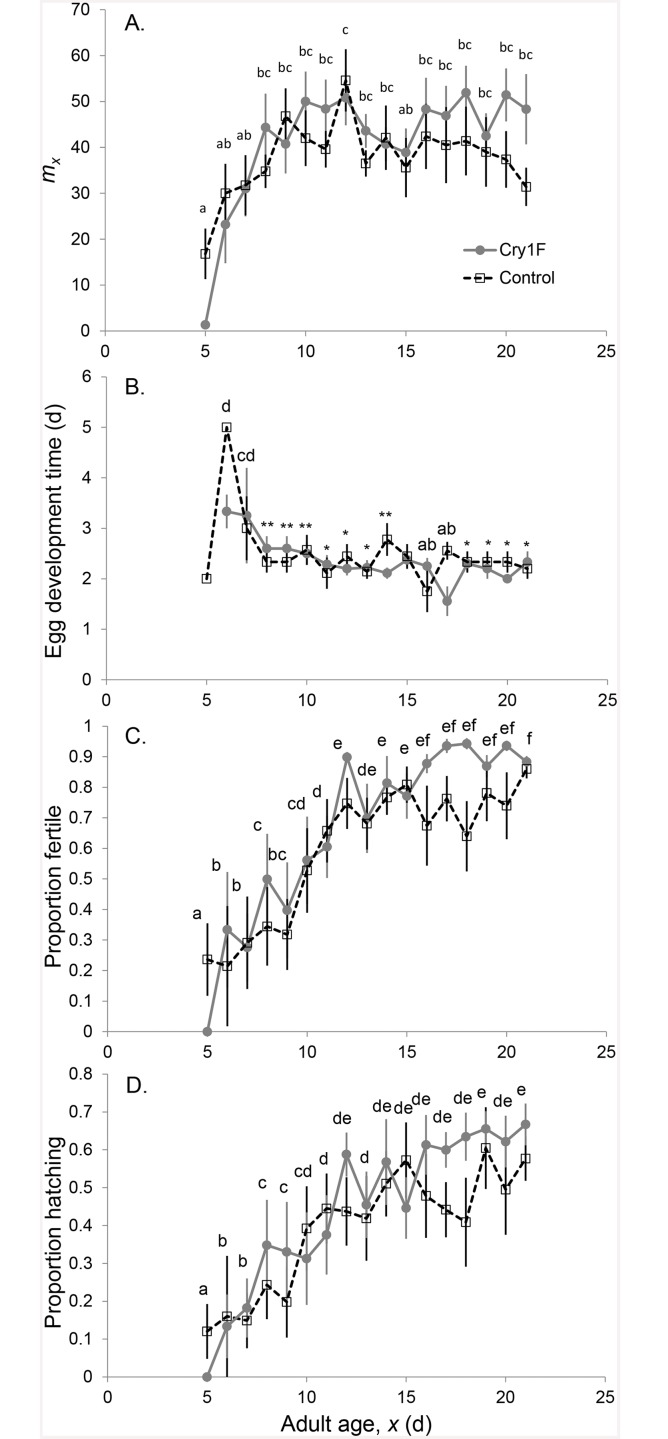
Average (±SE) age-specific reproduction. A) Age-specific fecundity (*m*_*x*_); B) Egg development time; C) Fertility; D) Hatching rate. Age averages with different letters are significantly different (Tukey’s HSD). For egg development, * = abc, ** = bcd. For fertility and hatching rate, averages were separated by confidence intervals on the age-specific parameter estimates.

There was no detrimental effect of the Cry1F on any reproductive parameter (Table 1, and S3 File). Instead, parents exposed to Cry1F through the tritrophic system had higher age-specific fecundity and the eggs from parents exposed to Cry1F through the tritrophic system had higher age-specific fertility and hatching rates. The following reproductive parameters varied according to the age of the parents (Fig 3, and S3 File) respectively: age-specific fecundity (*P*_age*treatment_ = 0.0021), egg development time (*P*_age_ = 0.0061), fertility (*P*_age_ = 0.0001) and hatching rate (*P*_age_ = 0.0014). Age-specific fecundity was low on the day of first reproduction and increased during the next five days reaching a higher plateau in the Cry1F treatment than the control around age 10. Age-specific egg development time varied in the beginning of the reproductive period, but after the parental age 7, the egg development time was constant and shorter. Age-specific hatching rate started low and steadily increased, reaching a higher value in the Cry1F treatment than the control at age 16. Age-specific fertility also started low, increasing to a plateau around age 12, with a higher plateau in the Cry1F treatment after age 16.

**Table 1:** Reproductive parameters of *H*. *axyridis* parents exposed and not exposed to Cry1F during 20 days through a tritrophic system by *M*. *persicae*.

	Control			Cry1F (20 μg/ml)			*P*
	Average	SE	*n*	Average	SE	*n*	
Pre-oviposition period (d)	6.2	0.4	10	5.9	0.5	10	0.6676
Age-specific fecundity (*m*_*x*_)	38.39	1.56	150	42.80	1.57	150	0.0241
Number of eggs produced	575.8	53.6	10	628.4	20.1	10	0.4203
Egg development time (d)	2.39	0.08	112	2.29	0.06	122	0.2426
Fertility rate (%)	60.32	2.88	141	71.50	2.87	145	0.0047
Hatching rate (%)	38.75	2.31	145	47.26	2.48	147	0.0466

**Fig 4:**
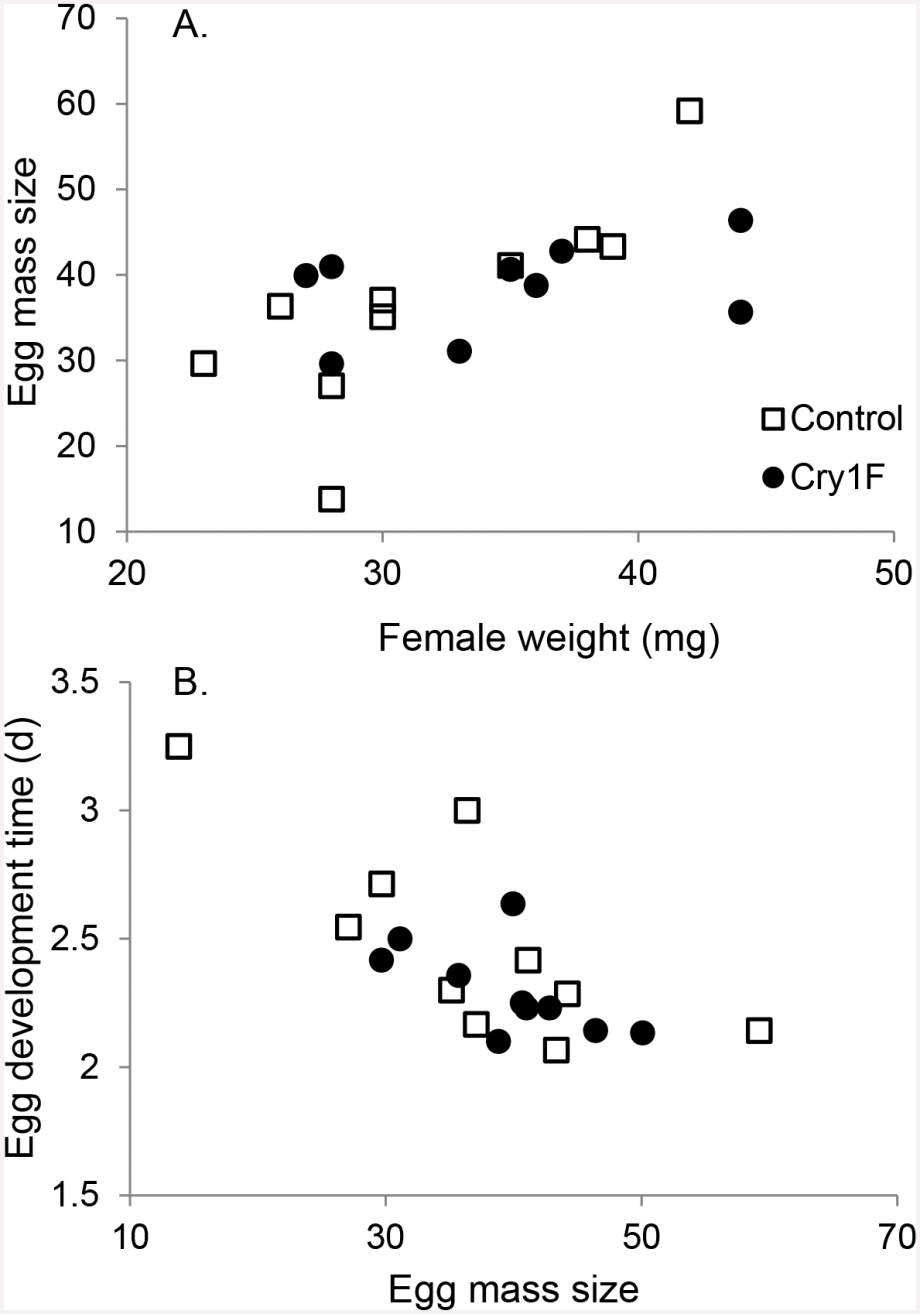
Relation between female weight with average age-specific fecundity and egg development time. Control showed significant correlations while the Cry1F treatment did not.

Females weighed 31.9±2.0 (SE) and 34.7±2.1 (SE) mg in the control and Cry1F treatments, respectively (*P* = 0.3554; S2 File). Males weighed respectively 23.6±0.9 (SE) and 24.1±1.8 (SE) mg (*P* = 0.8031). The bioassay was conducted in a growth chamber (25±2°C, 60±10% R.H. and 16L:8D). In the control treatment, mother weight was positively correlated with average age-specific fecundity (Fig 4A, *r* = 0.78, *n* = 10, *P* = 0.017; and S4 File). In addition, the higher was the average age-specific fecundity, the shorter was their egg development time (Fig 4B, *r* = -0.76, *n* = 10, *P* = 0.026). However, these correlations did not occur when the mother was exposed to Cry1F.

## Discussion

The detection of Cry1F in the eggs and larvae of *H*. *axyridis* after several days of parental exposure to the toxin confirmed that the adults are able to sequester Cry1F from their prey and transfer it to their offspring. In the mothers, the Cry1F probably was sequestered in the ovaries, where it could be transferred to the eggs during oogenesis. Cry1F was probably uptaken by the fathers, because it could be detected in them at concentrations similar to the mothers even 24 h after cessation of toxin exposure. Fathers could have transferred toxin to offspring via the spermatophore, however, this was not evaluated. In addition, it is not known if all the eggs or neonates in an egg batch had Cry1F from the parents, as they were not analyzed individually.

The Cry1F concentration in the eggs predicted the concentration in the neonates from the same egg batch, indicating that the toxin came from a common source. In addition, the positive correlation between the concentration of Cry1F in the parents (at the end of the experiment) and the concentration in all of their eggs indicates that the parents were the source of the Cry1F in the eggs. There was no such positive correlation between the parents and the neonates, but there were other sources of variation in Cry1F concentrations in the neonates. For example, there may be variation in uptake into the neonate from the egg. Although feeding on unhatched eggs or egg remnants could also contribute variation, these behaviors were not observed. Thus, three independent lines of evidence point to the fact that adult *H*. *axyridis* uptake and sequester Cry1F and transfer it to their eggs and neonate offspring.

Sequestration of Cry toxins and transfer to the offspring of different insect orders has been indicated periodically since 2006 [4–6]. In the case of an aphidophagous predator species related to the coccinellid studied in this work, Zhang *et al*. [4] observed that the aphidophagous coccinellid predator *Propylea japonica* acquired a Cry1Ab/Cry1Ac toxin through its prey (aphids) that fed on NuCOTN 33B cotton, and during the first 20 days the amount of toxin found in adults increased the longer the predator was exposed. They also detected the toxin in the neonates of these adults, which indicates sequestration and transfer to offspring. We found that the concentration of Cry1F toxin sequestered and transferred to the offspring increased in the eggs and neonates during the first five egg samples (first 12 days), paralleling the results of Zhang *et al*. [4], but it then leveled off and declined during the last five days of our experiment. Similar to our work, other studies also used detection of Cry toxin in offspring to demonstrate sequestering and transfer to offspring [5,6]. Coupled with the published literature, our results suggest that intergenerational transfer of Cry toxins may occur commonly, although more species and Cry toxins should be examined before this can be known conclusively.

The ecological implications of sequestration and transfer of a Cry toxin to an arthropod predator, found here and by Zhang *et al*. [4], is that this phenomenon might also happen in the field. In the field, exposure of predators to Cry toxins would be more variable than in a laboratory study. However, despite this potential limitation, the phenomenon is possible. To test this possibility, eggs of arthropod predators, including aphidophagous coccinellids, could be collected, carefully cleaned of all extraneous sources of Cry toxins adhering to the egg chorion, and checked by ELISA, especially in GM crops expressing Cry toxins in plant parts, from which prey of the predator could acquire the toxin, such as the GM cotton studied by Zhang *et al*. [4].

If Cry toxin is detected in field-collected predator eggs, two ecological implications would be: 1) a novel pathway (intergenerational transfer) for Cry toxin exposure at higher trophic levels, which has not been accounted for in current environmental risk assessments; 2) the longer persistence of Cry toxins in the food web, exposing natural enemies to Cry toxins with a higher likelihood than previously considered [6]. These would be more likely to affect species that have trophic interactions with these predators through cannibalism, intraguild predation and parasitism. However, even if sequester and transfer to offspring occurred in the field, the likelihood of ecological effects would remain to be determined.

We found that *H*. *axyridis* that fed on aphids exposed to Cry1F had higher age-specific fecundity, fertility and hatching rates compared to controls (Table 1). One possible explanation is that adults exposed to the Cry1F toxin consumed more aphids, similar to that observed by Nakasu *et al*. [23] for another predaceous coccinellid species. Whether these effects of Cry1F would occur in the field is unknown. For a different Cry toxin with confirmed toxicity to coccinellids, intergenerational transfer might affect the offspring and subsequently the population of the coccinellid, similar to the mortality observed in the offspring of a lepidopteran after intergenerational transfer of Cry1Ac [6].

## Supporting Information

S1 File Input datasets. (PDF)

S2 File Statistical analysis of Cry1F concentration in eggs, neonates, and adults of *H*. *axyridis*. (DOCX)

S3 File Analysis of reproductive parameters in *H*. *axyridis* in the control and Cry1F treatments. (DOCX)

S4 File Correlations among *H*. *axyridis* reproductive parameters. (DOCX)
