# Supplementary material for: Sequestration and Transfer of Cry Entomotoxin to the Eggs of a Predaceous Ladybird Beetle
Source: PLoS One. 2015 Dec 14;10(12):e0144895. doi: 10.1371/journal.pone.0144895 (PMC4682807; doi:10.1371/journal.pone.0144895)
Supplement: S1 File — (PDF) [file pone.0144895.s001.pdf]

Reproductive Parameters

Data repro;

Input trt $ couple Fwt Mwt alpha last totalegg; (* trt=treatment[C=control; F=Cry1F], couple=beetle couple number, Fwt=female weight, Mwt=male weight, alpha=age of first reproduction, last=age at oviposition of egg batch 15, totalegg=total number of eggs oviposited in the first 15 egg batches *)

Cards;

C	01	0.028	0.024	5	20	433
C	03	0.023	0.024	7	21	445
C	04	0.042	0.029	8	22	888
C	05	0.03	0.026	7	21	556
C	07	0.028	0.021	8	27	276
C	08	0.035	0.023	5	20	658
C	09	0.038	0.022	5	20	707
C	10	0.026	0.019	5	21	618
C	11	0.03	0.025	5	19	527
C	12	0.039	0.023	7	21	650
F	02	0.028	0.025	4	20	504
F	03	0.027	0.016	5	21	679
F	04	0.033	0.019	3	22	622
F	05	0.035	0.031	6	20	610
F	06	.	0.018	7	21	751
F	07	0.037	0.025	9	23	642
F	08	0.036	0.028	7	21	582
F	09	0.044	0.021	7	25	678
F	10	0.028	0.025	6	21	656
F	11	0.044	0.033	5	19	696

Data eggs;

Input Trt $ EM $ Couple Day Eggs eggs_left eggs_fertile eggs_hatched;

(* Trt=treatment [Control and F20=Cry1F, EM=egg batch, couple=beetle couple number, day=age of adults when egg batch was laid, eggs=number of eggs in egg batch, eggs_left=number of eggs that were left to develop, eggs_fertile=number of eggs_left that developed an embryo, eggs_hatched=number of eggs_left that hatched *)

If eggs_left = -99 then eggs_left = .;

If eggs_hatched = -99 then eggs_hatched = .;

If eggs_fertile =-99 then eggs_fertile = .;

Hatch = eggs_hatched/ eggs_left;

Fertile = eggs_fertile/eggs_left;

Cards;

Control	EM01	1	5	11	0	-99	-99
Control	EM02	1	6	54	28	2	0
Control	EM03	1	7	15	7	1	1
Control	EM04	1	8	42	21	21	15
Control	EM05	1	9	39	21	21	18
Control	EM06	1	10	20	11	11	9
Control	EM07	1	11	20	10	9	9
Control	EM08	1	12	36	22	18	16
Control	EM09	1	13	39	22	4	4
Control	EM10	1	14	22	13	10	9
Control	EM11	1	15	37	19	18	16
Control	EM12	1	16	39	-99	-99	-99
Control	EM13	1	17	17	10	9	6
Control	EM14	1	19	11	6	6	6
Control	EM15	1	20	31	18	17	16
Control	EM01	2	7	29	17	2	2
Control	EM02	2	8	25	13	6	4
Control	EM03	2	9	31	16	6	1
Control	EM04	2	10	31	17	11	6
Control	EM05	2	11	31	16	14	9
Control	EM06	2	12	32	20	1	0
Control	EM07	2	13	28	26	26	26
Control	EM08	2	14	27	15	14	13
Control	EM09	2	15	20	9	7	6

Control	EM10	2	16	21	11	11	11
Control	EM11	2	17	23	11	8	1
Control	EM12	2	18	49	25	24	19
Control	EM13	2	19	44	22	18	18
Control	EM14	2	20	32	17	14	12
Control	EM15	2	21	22	10	9	6
Control	EM01	3	8	37	19	13	12
Control	EM02	3	9	82	37	12	6
Control	EM03	3	10	61	25	20	17
Control	EM04	3	11	30	15	8	4
Control	EM05	3	12	94	53	39	24
Control	EM06	3	13	43	23	14	8
Control	EM07	3	14	83	41	25	10
Control	EM08	3	15	67	26	23	14
Control	EM09	3	16	49	26	20	9
Control	EM10	3	17	91	53	48	31
Control	EM11	3	18	82	43	38	23
Control	EM12	3	19	26	14	12	10
Control	EM13	3	20	69	29	1	0
Control	EM14	3	21	22	12	11	8
Control	EM15	3	22	52	37	32	20
Control	EM01	4	7	12	6	-99	0
Control	EM02	4	8	29	15	0	0
Control	EM03	4	9	31	20	0	0
Control	EM04	4	10	62	29	7	3
Control	EM05	4	11	41	22	18	14
Control	EM06	4	12	32	19	18	13
Control	EM07	4	13	46	18	16	15
Control	EM08	4	14	41	22	20	18
Control	EM09	4	15	21	10	10	10
Control	EM10	4	16	33	18	17	16
Control	EM11	4	17	60	29	11	10
Control	EM12	4	18	61	33	20	13
Control	EM13	4	19	20	10	10	7
Control	EM14	4	20	25	13	10	8
Control	EM15	4	21	42	10	9	6
Control	EM01	5	8	19	10	0	0
Control	EM02	5	9	27	15	0	0
Control	EM03	5	10	35	16	0	0
Control	EM04	5	11	38	18	13	4
Control	EM05	5	12	37	19	14	0
Control	EM06	5	13	33	17	6	3
Control	EM07	5	14	6	3	2	1
Control	EM08	5	15	8	4	2	0
Control	EM09	5	16	6	3	0	0
Control	EM10	5	17	3	0	-99	-99
Control	EM11	5	20	13	6	6	0
Control	EM12	5	23	21	9	3	0
Control	EM13	5	25	10	5	2	0
Control	EM14	5	26	15	9	8	4
Control	EM15	5	27	5	2	0	0
Control	EM01	6	5	16	6	0	0
Control	EM02	6	6	28	14	0	0
Control	EM03	6	7	46	26	1	1
Control	EM04	6	8	48	28	1	1
Control	EM05	6	9	72	43	20	15
Control	EM06	6	10	69	30	21	11
Control	EM07	6	11	47	24	22	14
Control	EM08	6	12	45	6	5	2
Control	EM09	6	13	48	27	26	20
Control	EM10	6	14	63	33	-99	6
Control	EM11	6	15	26	13	12	9
Control	EM12	6	16	30	17	17	11
Control	EM13	6	17	45	16	7	3
Control	EM14	6	18	20	10	2	0
Control	EM15	6	20	55	14	12	8
Control	EM01	7	5	34	8	3	2
Control	EM02	7	6	20	10	10	8
Control	EM03	7	7	57	32	30	19
Control	EM04	7	8	52	28	9	6
Control	EM05	7	9	64	33	1	1
Control	EM06	7	11	58	23	-99	16
Control	EM07	7	12	70	36	34	20
Control	EM08	7	13	30	14	7	0
Control	EM09	7	14	31	15	15	10
Control	EM10	7	15	57	29	26	19
Control	EM11	7	16	56	30	28	15
Control	EM12	7	17	63	27	24	18
Control	EM13	7	18	27	16	13	8
Control	EM14	7	19	51	18	5	3
Control	EM15	7	20	37	27	24	21
Control	EM01	8	5	22	9	3	1
Control	EM02	8	6	30	15	0	0
Control	EM03	8	7	56	35	0	0

Control	EM04	8	8	28	13	0	0
Control	EM05	8	9	34	14	1	0
Control	EM06	8	10	21	11	0	0
Control	EM07	8	11	42	21	1	0
Control	EM08	8	12	75	40	36	23
Control	EM09	8	13	22	12	9	0
Control	EM10	8	14	52	32	-99	-99
Control	EM11	8	15	65	33	15	7
Control	EM12	8	16	76	35	32	19
Control	EM13	8	17	35	13	12	6
Control	EM14	8	18	28	15	3	0
Control	EM15	8	21	32	6	5	4
Control	EM01	9	5	1	0	-99	-99
Control	EM02	9	6	18	9	0	0
Control	EM03	9	7	19	9	0	0
Control	EM04	9	8	23	9	0	0
Control	EM05	9	9	43	23	1	0
Control	EM06	9	10	44	24	-99	21
Control	EM07	9	11	30	15	4	1
Control	EM08	9	12	66	29	25	23
Control	EM09	9	13	30	17	13	10
Control	EM10	9	14	42	10	6	5
Control	EM11	9	15	27	12	11	10
Control	EM12	9	16	76	39	12	7
Control	EM13	9	17	21	7	7	5
Control	EM14	9	18	28	10	10	9
Control	EM15	9	19	59	20	15	9
Control	EM01	10	7	20	10	8	3
Control	EM02	10	8	45	17	16	9
Control	EM03	10	9	45	23	20	11
Control	EM04	10	10	35	18	15	6
Control	EM05	10	11	59	31	26	16
Control	EM06	10	12	59	28	18	7
Control	EM07	10	13	46	25	20	8
Control	EM08	10	14	54	31	20	9
Control	EM09	10	15	28	14	11	4
Control	EM10	10	16	38	10	2	2
Control	EM11	10	17	47	37	26	12
Control	EM12	10	18	36	11	5	2
Control	EM13	10	19	62	34	26	13
Control	EM14	10	20	37	20	12	8
Control	EM15	10	21	39	20	15	7

F20	EM01	1	4	3	3	0	0
F20	EM02	1	5	1	1	0	0
F20	EM03	1	7	45	25	8	8
F20	EM04	1	8	65	30	30	21
F20	EM05	1	9	33	15	13	10
F20	EM06	1	10	42	19	19	18
F20	EM07	1	11	50	28	28	23
F20	EM08	1	12	57	42	41	33
F20	EM09	1	13	38	22	0	0
F20	EM10	1	14	32	16	12	8
F20	EM11	1	15	50	30	27	24
F20	EM12	1	16	17	11	9	3
F20	EM13	1	17	24	12	11	11
F20	EM14	1	18	26	14	14	8
F20	EM15	1	19	21	12	8	5
F20	EM01	2	5	1	1	0	0
F20	EM02	2	6	49	22	17	3
F20	EM03	2	7	27	10	0	0
F20	EM04	2	8	53	23	4	0
F20	EM05	2	9	57	26	26	25
F20	EM06	2	10	54	32	29	3
F20	EM07	2	11	74	33	24	4
F20	EM08	2	12	49	26	23	20
F20	EM09	2	13	63	33	31	24
F20	EM10	2	14	48	28	5	0
F20	EM11	2	16	19	12	12	11
F20	EM12	2	18	50	27	27	22
F20	EM13	2	19	44	23	19	13
F20	EM14	2	20	40	22	20	16
F20	EM15	2	21	51	21	18	18
F20	EM01	3	3	1	1	1	0
F20	EM02	3	6	2	3	0	0
F20	EM03	3	7	13	7	-99	-99
F20	EM04	3	8	76	43	43	37
F20	EM05	3	10	84	43	31	3
F20	EM06	3	11	34	18	10	10
F20	EM07	3	12	99	61	59	40
F20	EM08	3	13	33	16	2	2
F20	EM09	3	14	43	23	22	18
F20	EM10	3	15	44	21	18	7
F20	EM11	3	17	38	21	19	10
F20	EM12	3	18	29	16	14	6

F20	EM13	3	19	67	37	31	20
F20	EM14	3	21	35	18	16	14
F20	EM15	3	22	24	12	12	9
F20	EM01	4	6	32	22	18	10
F20	EM02	4	7	53	27	-99	-99
F20	EM03	4	8	27	14	0	0
F20	EM04	4	9	45	27	0	0
F20	EM05	4	10	30	18	0	0
F20	EM06	4	11	38	14	1	0
F20	EM07	4	12	52	25	22	6
F20	EM08	4	13	25	14	13	9
F20	EM09	4	14	50	18	8	0
F20	EM10	4	15	12	4	1	0
F20	EM11	4	16	55	27	26	22
F20	EM12	4	17	29	15	15	10
F20	EM13	4	18	68	37	32	18
F20	EM14	4	19	37	12	12	9
F20	EM15	4	20	57	31	28	17
F20	EM01	5	7	14	7	4	3
F20	EM02	5	8	42	22	19	16
F20	EM03	5	9	31	10	2	2
F20	EM04	5	10	62	26	20	13
F20	EM05	5	11	75	43	20	16
F20	EM06	5	12	43	17	15	10
F20	EM07	5	13	38	12	11	5
F20	EM08	5	14	29	14	14	14
F20	EM09	5	15	46	12	7	4
F20	EM10	5	16	56	31	24	13
F20	EM11	5	17	81	45	-99	24
F20	EM12	5	18	71	34	33	21
F20	EM13	5	19	35	20	14	11
F20	EM14	5	20	55	35	34	21
F20	EM15	5	21	73	15	14	10
F20	EM01	6	9	5	2	0	0
F20	EM02	6	10	60	29	0	0
F20	EM03	6	11	55	25	19	10
F20	EM04	6	12	43	22	20	11
F20	EM05	6	13	53	30	25	16
F20	EM06	6	14	22	13	13	6
F20	EM07	6	15	57	21	20	11
F20	EM08	6	16	37	20	18	14
F20	EM09	6	17	49	20	20	13
F20	EM10	6	18	83	51	45	30
F20	EM11	6	19	23	12	12	7
F20	EM12	6	20	65	35	34	25
F20	EM13	6	21	20	11	-99	6
F20	EM14	6	22	37	15	15	11
F20	EM15	6	23	33	6	5	4
F20	EM01	7	7	14	7	0	0
F20	EM02	7	8	16	6	0	0
F20	EM03	7	9	48	27	0	0
F20	EM04	7	10	20	11	0	0
F20	EM05	7	11	31	15	2	0
F20	EM06	7	12	28	16	13	13
F20	EM07	7	13	51	18	18	15
F20	EM08	7	14	36	19	19	17
F20	EM09	7	15	34	18	15	5
F20	EM10	7	16	54	27	26	20
F20	EM11	7	17	65	34	31	19
F20	EM12	7	18	47	19	19	19
F20	EM13	7	19	34	20	18	18
F20	EM14	7	20	43	22	22	20
F20	EM15	7	21	61	29	24	19
F20	EM01	8	7	52	27	4	1
F20	EM02	8	8	64	38	28	14
F20	EM03	8	11	12	6	3	0
F20	EM04	8	12	49	27	24	10
F20	EM05	8	13	55	23	14	5
F20	EM06	8	14	39	22	20	9
F20	EM07	8	15	31	10	9	6
F20	EM08	8	16	78	47	38	19
F20	EM09	8	17	28	15	15	8
F20	EM10	8	18	54	27	25	10
F20	EM11	8	19	45	16	15	12
F20	EM12	8	20	28	17	15	6
F20	EM13	8	23	44	33	8	4
F20	EM14	8	24	57	23	20	5
F20	EM15	8	25	42	25	25	11
F20	EM01	9	6	8	4	0	0
F20	EM02	9	8	14	5	0	0
F20	EM03	9	9	43	19	4	4
F20	EM04	9	10	37	19	17	15
F20	EM05	9	11	49	28	26	22
F20	EM06	9	12	36	22	21	13

F20	EM07	9	13	37	19	13	7
F20	EM08	9	14	62	35	33	30
F20	EM09	9	15	20	9	7	4
F20	EM10	9	16	67	31	23	14
F20	EM11	9	17	64	32	26	14
F20	EM12	9	18	35	16	16	12
F20	EM13	9	19	62	32	28	25
F20	EM14	9	20	72	58	53	29
F20	EM15	9	21	50	22	20	11
F20	EM01	10	5	2	0	-99	-99
F20	EM02	10	6	25	13	1	1
F20	EM03	10	7	30	13	8	4
F20	EM04	10	8	42	21	15	10
F20	EM05	10	9	64	30	27	17
F20	EM06	10	10	61	29	22	12
F20	EM07	10	11	66	33	30	23
F20	EM08	10	12	52	23	19	13
F20	EM09	10	13	43	19	18	13
F20	EM10	10	14	46	22	21	17
F20	EM11	10	15	56	27	24	19
F20	EM12	10	16	52	14	13	10
F20	EM13	10	17	44	32	30	20
F20	EM14	10	18	56	22	20	17
F20	EM15	10	19	57	39	37	28

Cry1F Concentrations (determined by ELISA)

Data egg;

Input couple em FEconc; (* nB, all are from couples in Cry1F treatment, couple=beetle couple number, em=egg sample number, FEconc=concentration of Cry1F in egg samples (ng/egg), samples below the LOD were 0 *)

Cards;

2	1	28.9295748
2	2	2.654707728
2	3	13.17613267
2	4	19.7963052
2	5	32.81676429
2	6	0
2	7	14.22626734
2	8	37.67826948
2	9	1.577126277
2	10	0
3	1	0
3	2	2.829310321
3	3	6.887994
3	4	25.55327307
3	5	0.522556734
3	6	12.95346651
3	7	5.475983436
3	8	79.11109325
3	9	12.63740409
3	10	0
4	1	0
4	2	4.935361467
4	3	2.76518678
4	4	17.0710554
4	5	9.807187701
4	6	31.53865925
4	7	5.2889604
4	8	2.7769165
4	9	4.738978808
4	10	0
5	1	2.177000329
5	2	3.421531154
5	3	11.293828
5	4	14.0171892
5	5	29.54785523
5	6	17.4511633
5	7	1.498414139
5	8	7.766152121
5	9	12.98417896
5	10	5.320360326
6	1	4.926687252
6	2	6.448100089
6	3	3.909402
6	4	8.032044109
6	5	0
6	6	0
6	7	7.824532062
6	8	24.3748112
6	9	2.015748719
6	10	1.710621933
7	1	0
7	2	1.997762715
7	3	2.359729135
7	4	24.9091879
7	5	12.77836983
7	6	8.627143686
7	7	1.160127736
7	8	26.76854425
7	9	20.64421665
7	10	23.72844671
8	1	0
8	2	2.515836089
8	3	0
8	4	15.23798024
8	5	5.539224454
8	6	0

8	7	10.74755263
8	8	7.519565822
8	9	3.317435702
8	10	3.507719118
9	1	0.725110206
9	2	4.549328805
9	3	4.97797188
9	4	12.70121272
9	5	43.61372309
9	6	5.1742592
9	7	1.004693044
9	8	15.10670156
9	9	7.368981449
9	10	4.174564474
10	1	0
10	2	3.867625755
10	3	5.598649778
10	4	12.5440812
10	5	50.97135362
10	6	29.00560452
10	7	4.0480792
10	8	22.77009476
10	9	0
10	10	0
11	1	0
11	2	8.664500427
11	3	3.992580766
11	4	4.558309543
11	5	7.78622565
11	6	20.61924923
11	7	0.2135964
11	8	0
11	9	1.461440649
11	10	3.989500071

Data neonate;
Input couple nl FLconc; (* nB, all are from couples in Cry1F treatment, couple=beetle couple number, nl=neonate larva sample number, FLconc=concentration of Cry1F in neonate samples (ng/larva), samples below the LOD were 0 *)
Cards;

3	1	0
5	1	0.613456439
6	1	1.739206218
9	1	22.1595669
2	2	0.913700416
6	2	0
7	2	8.562958001
10	2	0
11	2	14.7722521
2	3	0
4	3	0
6	3	23.07854028
7	3	14.28631748
9	3	21.05379014
10	3	18.67992725
11	3	8.884268793
3	4	0
4	4	0
6	4	4.097194813
8	4	17.02889476
9	4	8.22932307
2	5	16.82718844
6	5	61.25792784
7	5	27.61280352
8	5	10.74678764
9	5	52.82900417
10	5	51.51553937
11	5	34.5224984
2	6	21.34877599
3	6	23.07741274
4	6	64.2499672
5	6	0
6	6	0
7	6	33.38332811
9	6	0
10	6	20.38729773
11	6	51.40879101
2	7	131.3603758
3	7	20.05013038
4	7	71.39202776
6	7	0
7	7	7.351787855
8	7	1.874121775
9	7	2.815449481
10	7	11.91852686
11	7	0.365021305
2	8	4.24010426
4	8	0
6	8	0
7	8	0
8	8	5.146895389
10	8	5.691516105
11	8	0
2	9	0
3	9	0
4	9	0
5	9	0
6	9	5.105324175
7	9	0
8	9	13.06505621
9	9	90.91651967
10	9	0
11	9	0
2	10	18.14854076
3	10	1.26582583
4	10	25.20464675
5	10	8.225899147
6	10	7.382137809
7	10	39.98288179
9	10	44.93993184
10	10	0
11	10	7.784294205
