# Supplementary material for: Sequestration and Transfer of Cry Entomotoxin to the Eggs of a Predaceous Ladybird Beetle
Source: PLoS One. 2015 Dec 14;10(12):e0144895. doi: 10.1371/journal.pone.0144895 (PMC4682807; doi:10.1371/journal.pone.0144895)
Supplement: S2 File — (DOCX) [file pone.0144895.s002.docx]

**S2. Statistical analysis of Cry1F concentration in eggs, neonates, and adults of *H. axyridis***

Eggs. ANOVA table for Cry1F egg concentrations in egg mass samples.

| **Source** | **DF** | **SS** | **MS** | ***F*** | ***P*** |
| --- | --- | --- | --- | --- | --- |
| **Couple** | 9 | 1322.2627 | 146.91807 | 1.627 | 0.1179 |
| **Egg mass order** | 9 | 4384.1273 | 487.12526 | 5.394 | 5.350E-06 |
| **Couple*Egg mass order** | 81 | 10230.46 | 126.3019 | 1.398 | 0.0558 |
| **Error** | 99 | 8940.9335 | 90.31246 |  |  |

Cry1F concentration in eggs varied with order that the egg masses were produced by the couples. There were not significant differences among Cry1F couples or the interaction of couple and egg mass order.

Tukey HSD separation of Cry1F concentration in eggs according to egg mass order.

| **Egg Mass Order** | **N** | **Mean (ng/egg)** | **Tukey Grouping** | |
| --- | --- | --- | --- | --- |
| 8 | 10 | 22.39 | A |  |
| 5 | 10 | 19.34 | A | B |
| 4 | 10 | 15.44 | A | B |
| 6 | 10 | 12.54 | C | B |
| 9 | 10 | 6.68 | C | D |
| 3 | 10 | 5.50 | C | D |
| 7 | 10 | 5.15 |  | D |
| 10 | 10 | 4.24 |  | D |
| 2 | 10 | 4.19 |  | D |
| 1 | 10 | 3.68 |  | D |

Neonates. ANOVA table for Cry1F neonate concentrations in neonate samples.

| **Source** | **DF** | **SS** | **MS** | ***F*** | ***P*** |
| --- | --- | --- | --- | --- | --- |
| **Couple** | 9 | 3923.14293 | 435.905 | 1.167 | 0.3291 |
| **Neonate order** | 9 | 7491.01626 | 832.335 | 2.229 | 0.0297 |
| **Couple*Neonate order** | 53 | 29457.3802 | 555.8 | 1.488 | 0.0587 |
| **Error** | 71 | 26510.6945 | 373.3901 |  |  |

Cry1F concentration in neonates varied with order that the egg masses from which the neonates emerged were produced by the couples. There were not significant differences among Cry1F couples or the interaction of couple and egg mass order.

Tukey HSD separation of Cry1F concentration neonates according to neonate order.

| **Neonate Order** | **N** | **Mean (ng/neonate)** | **Tukey Grouping** | |
| --- | --- | --- | --- | --- |
| 5 | 7 | 36.47 | A |  |
| 7 | 9 | 27.46 | A |  |
| 6 | 9 | 23.76 | A |  |
| 10 | 9 | 16.99 | A | B |
| 3 | 7 | 12.28 | A | B |
| 9 | 10 | 10.91 | A | B |
| 1 | 4 | 6.13 | A | B |
| 4 | 5 | 5.87 | A | B |
| 2 | 5 | 4.85 | A | B |
| 8 | 7 | 2.15 |  | B |

Neonates. ANCOVA table for Cry1F concentrations in neonate samples using Cry1F concentrations in egg masses as the covariate to predict neonate concentrations.

| **Source** | **DF** | **SS** | **MS** | ***F*** | ***P*** |
| --- | --- | --- | --- | --- | --- |
| **Egg concentration** | 1 | 1859.66 | 1859.6619 | 4.980 | 0.0300 |
| **Couple** | 9 | 3881.41 | 431.26794 | 1.155 | 0.3429 |
| **Neonate order** | 9 | 7087.48 | 787.49771 | 2.109 | 0.0453 |
| **Couple*Neonate order** | 52 | 27597.7 | 530.72535 | 1.421 | 0.1041 |
| **Error** | 71 | 26510.7 | 373.3900632 |  |  |

Cry1F concentration in neonates was predicted by the concentration in the egg mass from which they came, and still varied with order of the egg masses from which the neonates emerged that were produced by the couples. There were not significant differences among Cry1F couples or the interaction of couple and egg mass order.

Parameter estimates for the covariate, Cry1F egg concentration.

| **Parameter** | **Estimate** | **SE** | ***t*** | ***P*** |
| --- | --- | --- | --- | --- |
| **Intercept** | 15.58 | 11.01 | 1.415 | 0.1614 |
| **Egg concentration** | 0.591 | 0.267 | 2.215 | 0.0300 |

This means that on average the neonate concentration is 59% of the egg concentration.

Adults. ANOVA table for Cry1F adult concentrations per mg F.W.

| **Source** | **DF** | **SS** | **MS** | ***F*** | ***P*** |
| --- | --- | --- | --- | --- | --- |
| **Sex** | 1 | 6.193853 | 6.193853 | 0.02604 | 0.8736 |
| **Error** | 18 | 4281.456 | 237.8586 |  |  |

Average concentrations in adults by sex.

| **Sex** | ***N*** | **Mean (ng/mg FW)** | **SE** |
| --- | --- | --- | --- |
| **Female** | 10 | 11.46 | 2.01 |
| **Male** | 10 | 10.35 | 0.85 |
