# Supplementary material for: Sequestration and Transfer of Cry Entomotoxin to the Eggs of a Predaceous Ladybird Beetle
Source: PLoS One. 2015 Dec 14;10(12):e0144895. doi: 10.1371/journal.pone.0144895 (PMC4682807; doi:10.1371/journal.pone.0144895)
Supplement: S3 File — (DOCX) [file pone.0144895.s003.docx]

**S3. Analysis of reproductive parameters in *H. axyridis* in the control and Cry1F treatments**

Age-specific fecundity (*m_x_*). Comparison of repeated measures models using AIC_c_ (smaller is better) with differing correlation structures among ages.

| **Correlation** |  | **AIC_c_** |
| --- | --- | --- |
| Independent |  | 2885.8 |
| Banded Toeplitz-1 |  | 2875.5 |
| AR-1 |  | 2869.2 |

The model with AR-1 correlation among ages is the best model.

Age-specific fecundity (*m_x_*). Repeated measures ANOVA table with AR-1 correlation among ages for daily fecundity of *H. axyridis*.

| **Source** | **Num DF** | **Den DF** | ***F*** | ***P*** |
| --- | --- | --- | --- | --- |
| **Treatment** | 1 | 18 | 6.06 | 0.0241 |
| **Age** | 1 | 301 | 1.78 | 0.1826 |
| **Treatment*Age** | 1 | 301 | 9.66 | 0.0021 |

Significant difference between treatments.

Average daily fecundity for control and Cry1F diet.

| **Treatment** | ***N*** | **Daily fecundity** | |
| --- | --- | --- | --- |
|  |  | **Mean** | **SE** |
| **Control** | **150** | 38.4 | 1.562 |
| **F20** | **150** | 42.8 | 1.566 |

Egg development time. Comparison of repeated measures models using AIC_c_ (smaller is better) with differing correlation structures among ages.

| **Correlation** |  | **AIC_c_** |
| --- | --- | --- |
| Independent |  | 517.1 |
| Banded Toeplitz-1 |  | 519.2 |
| AR-1 |  | 519.2 |

The model with no correlation among ages is the best model.

Egg development time. Repeated measures ANOVA table with no correlation among ages for age-specific development time of *H. axyridis* eggs.

| **Source** | **Num DF** | **Den DF** | ***F*** | ***P*** |
| --- | --- | --- | --- | --- |
| **Treatment** | 1 | 18 | 1.46 | 0.2426 |
| **Age** | 1 | 301 | 7.66 | 0.0061 |
| **Treatment*Age** | 1 | 301 | 0.89 | 0.3478 |

No significant difference between control and Cry1F.

Average egg development time for control and Cry1F diet.

| **Treatment** | ***N*** | **Development Time** | |
| --- | --- | --- | --- |
|  |  | **Mean** | **SE** |
| **Control** | **112** | 2.393 | 0.0754 |
| **F20** | **122** | 2.295 | 0.0601 |

Fertility rate. Comparison of repeated measures models using QIC (smaller is better) with differing correlation structures among ages and scale parameters (no scale or scale based on estimated Pearson residual deviance).

| **Correlation** | **Scale** | **QIC** |
| --- | --- | --- |
| Independent | 1 | 392.367 |
| 1-m-dependent | 1 | 396.620 |
| AR-1 | 1 | 336.649 |
| Independent | Pearson | 45.902 |
| 1-m-dependent | Pearson | 46.480 |
| AR-1 | Pearson | 46.390 |

The model with no correlation among ages and a scale parameter based on the estimated Pearson residual deviance is the best model.

Fertility rate. Score statistics for type 3 generalized estimating equation logistic regression analysis for age-specific fertility rate of *H. axyridis*.

| **Source** | **DF** | ***Χ^2^*** | ***P*** |
| --- | --- | --- | --- |
| **Treatment** | 1 | 7.98 | 0.0047 |
| **Age** | 1 | 14.39 | 0.0001 |

Significant difference between control and Cry1F.

Average fertility rate for control and Cry1F diet.

| **Treatment** | ***N*** | **Fertility Rate** | |
| --- | --- | --- | --- |
|  |  | **Mean** | **SE** |
| **Control** | **141** | 0.603 | 0.029 |
| **F20** | **145** | 0.715 | 0.029 |

Hatching rate. Comparison of repeated measures models using QIC (smaller is better) with differing correlation structures among ages and scale parameters (no scale or scale based on Pearson residual deviance).

| **Correlation** | **Scale** | **QIC** |
| --- | --- | --- |
| Independent | 1 | 462.28 |
| 1-m-dependent | 1 | 463.13 |
| AR-1 | 1 | 462.80 |
| Independent | Pearson | 76.1861 |
| 1-m-dependent | Pearson | 76.3254 |
| AR-1 | Pearson | 76.2715 |

The model with no correlation among ages and a scale parameter based on the estimated Pearson residual deviance is the best model.

Hatching rate. Score statistics for type 3 generalized estimating equation logistic regression analysis for age-specific fertility rate of *H. axyridis*.

| **Source** | **DF** | ***Χ^2^*** | ***P*** |
| --- | --- | --- | --- |
| **Treatment** | 1 | 3.96 | 0.0466 |
| **Age** | 1 | 10.20 | 0.0014 |

Significant difference between control and Cry1F.

Average hatching rate for control and Cry1F diet.

| **Treatment** | ***N*** | **Hatching Rate** | |
| --- | --- | --- | --- |
|  |  | **Mean** | **SE** |
| **Control** | **145** | 0.387 | 0.023 |
| **F20** | **147** | 0.473 | 0.025 |

Female weight. ANOVA table for female weight of *H. axyridis*.

| **Source** | **DF** | **SS** | **MS** | ***F*** | ***P*** |
| --- | --- | --- | --- | --- | --- |
| **Treatment** | 1 | 3.63E-05 | 0.00003626 | 0.900 | 0.3554 |
| **Error** | 17 | 0.000683 | 0.00004017 |  |  |

Male weight. ANOVA table for male weight of *H. axyridis*.

| **Source** | **DF** | **SS** | **MS** | ***F*** | ***P*** |
| --- | --- | --- | --- | --- | --- |
| **Treatment** | 1 | 1.25E-06 | 1.25E-06 | 0.060 | 0.8031 |
| **Error** | 18 | 0.0003513 | 1.952E-05 |  |  |

Age of first reproduction. ANOVA table for age of first reproduction of *H. axyridis*.

| **Source** | **DF** | **SS** | **MS** | ***F*** | ***P*** |
| --- | --- | --- | --- | --- | --- |
| **Treatment** | 1 | 0.4500 | 0.4500 | 0.190 | 0.6676 |
| **Error** | 18 | 42.500 | 2.36111111 |  |  |

Total eggs. ANOVA table for total eggs laid by *H. axyridis* in its first 15 egg masses.

| **Source** | **DF** | **SS** | **MS** | ***F*** | ***P*** |
| --- | --- | --- | --- | --- | --- |
| **Treatment** | 1 | 12285.025 | 12285.025 | 0.680 | 0.4203 |
| **Error** | 16 | 287239.475 | 17952.4672 |  |  |
