# Supplementary material for: Sequestration and Transfer of Cry Entomotoxin to the Eggs of a Predaceous Ladybird Beetle
Source: PLoS One. 2015 Dec 14;10(12):e0144895. doi: 10.1371/journal.pone.0144895 (PMC4682807; doi:10.1371/journal.pone.0144895)
Supplement: S4 File — (DOCX) [file pone.0144895.s004.docx]

**S4. Correlations among *H. axyridis* reproductive parameters**

Control couples. Pearson correlation coefficients (*r*) with *P*-value underneath. *N*=10 for all cases.

|  | Female weight | Male weight | Age of first reproduction (α) | Total eggs | Eggs/ day | Egg development time | Hatching rate |
| --- | --- | --- | --- | --- | --- | --- | --- |
| Male weight | 0.410 |  |  |  |  |  |  |
|  | 0.410 |  |  |  |  |  |  |
| Age of first reproduction (*α*) | 0.151 | 0.392 |  |  |  |  |  |
|  | 0.736 | 0.438 |  |  |  |  |  |
| Total eggs | 0.788 | 0.414 | -0.058 |  |  |  |  |
|  | 0.015 | 0.405 | 0.789 |  |  |  |  |
| Eggs/day | 0.782 | 0.524 | -0.008 | 0.987 |  |  |  |
|  | 0.017 | 0.244 | 0.798 | 0.000 |  |  |  |
| Egg Development Time | -0.691 | -0.670 | 0.045 | -0.670 | -0.757 |  |  |
|  | 0.064 | 0.080 | 0.792 | 0.080 | 0.026 |  |  |
| Hatching rate | 0.044 | 0.596 | -0.195 | 0.170 | 0.270 | -0.588 |  |
|  | 0.792 | 0.153 | 0.696 | 0.720 | 0.610 | 0.162 |  |
| Fertility rate | 0.361 | 0.579 | 0.124 | 0.316 | 0.410 | -0.686 | 0.850 |
|  | 0.484 | 0.173 | 0.755 | 0.548 | 0.410 | 0.068 | 0.003 |

Cry1F couples. Pearson correlation coefficients (*r*) with *P*-value underneath. *N*=10 for all cases.

|  | Female weight | Male weight | Age of first reproduction (α) | Total eggs | Eggs/ day | Egg development time | Hatching rate |
| --- | --- | --- | --- | --- | --- | --- | --- |
| Male weight | 0.432 |  |  |  |  |  |  |
|  | 0.378 |  |  |  |  |  |  |
| Age of first reproduction (*α*) | 0.373 | 0.116 |  |  |  |  |  |
|  | 0.466 | 0.761 |  |  |  |  |  |
| Total eggs | 0.431 | -0.308 | 0.312 |  |  |  |  |
|  | 0.379 | 0.559 | 0.555 |  |  |  |  |
| Eggs/day | 0.366 | 0.154 | 0.548 | 0.767 |  |  |  |
|  | 0.476 | 0.734 | 0.212 | 0.022 |  |  |  |
| Egg Development Time | -0.512 | -0.618 | -0.563 | -0.183 | -0.606 |  |  |
|  | 0.260 | 0.129 | 0.193 | 0.708 | 0.142 |  |  |
| Hatching rate | -0.100 | 0.095 | -0.074 | 0.083 | 0.312 | -0.315 |  |
|  | 0.770 | 0.773 | 0.783 | 0.779 | 0.554 | 0.550 |  |
| Fertility rate | 0.440 | -0.204 | 0.072 | 0.513 | 0.235 | -0.052 | 0.473 |
|  | 0.365 | 0.687 | 0.784 | 0.259 | 0.652 | 0.790 | 0.316 |
